# Integrating multiple sign types to improve occupancy estimation for inconspicuous species

**DOI:** 10.1002/ece3.10019

**Published:** 2023-05-15

**Authors:** Mia R. Goldman, Matthew Shinderman, Mackenzie R. Jeffress, Thomas J. Rodhouse, Kevin T. Shoemaker

**Affiliations:** ^1^ Department of Natural Resources and Environmental Sciences University of Nevada – Reno Reno Nevada USA; ^2^ School of Biology University of St Andrews St Andrews UK; ^3^ Department of Biology and Center for Research into Environmental and Ecological Modeling University of St Andrews St Andrews UK; ^4^ Department of Natural Resources and Sustainability Oregon State University‐Cascades Bend Oregon USA; ^5^ Nevada Department of Wildlife Elko Nevada USA; ^6^ National Park Service Bend Oregon USA

**Keywords:** American pika, detection probability, false negative, false positive, indirect sign, multi‐sign, occupancy model, population dynamics

## Abstract

Standard occupancy models enable unbiased estimation of occupancy by accounting for observation errors such as missed detections (false negatives) and, less commonly, incorrect detections (false positives). Occupancy models are fitted to data from repeated site visits in which surveyors record evidence of species presence. Use of indirect sign (e.g., scat, tracks) as evidence of presence can vastly improve survey efficiency for inconspicuous species but can also introduce additional sources of error. We developed a “multi‐sign” occupancy approach to model the detection process separately for unique sign types and used this method to improve estimates of occupancy dynamics for an inconspicuous species, the American pika (*Ochotona princeps*). We investigated how estimates of pika occupancy and environmental drivers differed under four increasingly realistic representations of the observation process: (1) perfect detection (commonly assumed for modeling pika occupancy), (2) standard occupancy model (single observation process without possibility of false detection), (3) multi‐sign with no false detections (non‐false positive model), and (4) multi‐sign with false detections (full model). For the multi‐sign occupancy models, we modeled the detection of each sign type (fresh scat, fresh haypiles, pika calls, and pika sightings) separately as a function of climatic and environmental covariates. Estimates of occupancy processes and inferences about environmental drivers were sensitive to different detection models. Simplified representations of the detection processes generally resulted in higher occupancy estimates and higher turnover rates than the full multi‐sign model. Environmental drivers also varied in their influence on occupancy models, where (e.g.) forb cover was estimated to more strongly influence occupancy in the full multi‐sign model than the simpler models. As has been reported previously in other contexts, unmodeled heterogeneity in the observation process can lead to biases in occupancy processes and uncertainty in the relationships between occupancy and environmental covariates. Overall, our multi‐sign approach to dynamic occupancy modeling, which accounts for spatio‐temporal variation in reliability among sign types, has strong potential to generate more realistic estimates of occupancy dynamics for inconspicuous species.

## Graphical abstract

Standard occupancy models enable unbiased estimation of occupancy by accounting for observation errors such as missed detections (false negatives) and, less commonly, incorrect detections (false positives). We developed a “multi‐sign” occupancy approach to model the detection process separately for unique sign types and used this method to improve estimates of occupancy dynamics for an inconspicuous species, the American pika (*Ochotona princeps*). We demonstrated that a “multi‐sign” approach to dynamic occupancy modeling and the inclusion of false detection errors have strong potential to generate more robust estimates of occupancy dynamics and account for variation in sign‐type reliability for inconspicuous species than standard occupancy modeling approaches.

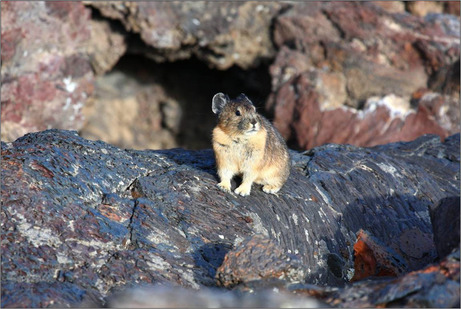

## 1 INTRODUCTION

Dynamic occupancy models have emerged as one of the most powerful tools to investigate species responses to global change at regional and range‐wide scales (MacKenzie, 2002; Nichols et al., 2008; Royle, 2006). These models use binary detection surveys to assess occupancy rates, changes, and drivers of occupancy. Because occupancy models rely on noninvasive survey designs that can be replicated over large areas at relatively low cost, this tool has gained popularity as research questions in conservation biology and ecology have increased in their geographic scope of inference. Occupancy modeling relies on direct (aural or visual) or indirect (e.g., scat, tracks) sign to determine the presence or absence of a species at each survey location. These models are used to advance important conservation and management goals across a wide range of taxa and geographic scales including investigating metapopulation dynamics of potentially climate‐sensitive species (Hanski & Gilpin, 1991; Moilanen et al., 1998), determining habitat use of a range of imperiled tropical avian species (Ruiz‐Gutie'rrez et al., 2010), and understanding the breeding status of potentially threatened amphibians in the Greater Yellowstone Area (Gould et al., 2019).

Most occupancy surveys are prone to detection errors, including failing to detect a species when it is present (false negative) or, perhaps less commonly, falsely detecting a species when it is in fact not present (false positive). If not correctly accounted for, detection errors can result in incorrect conclusions regarding occupancy processes (Berigan et al., 2019; MacKenzie et al., 2003; McClintock et al., 2010). Standard occupancy models are able to produce unbiased estimates of occupancy by explicitly modeling the observation process and treating occupancy status as an unobserved or partially observed latent process within a hierarchical modeling framework (Royle & Dorazio, 2008). To effectively tease apart the detection and occupancy processes, these models rely on surveyors conducting repeated visits to a site (one or more resurveys) within a time horizon short enough to assume no change in occupancy status (MacKenzie, 2002). In this framework, both the detection process and the occupancy process can be modeled separately as functions of distinct sets of covariates, thereby allowing researchers to control for detection errors when estimating the drivers of occupancy (MacKenzie et al., 2003).

In occupancy modeling, many analyses lack sufficient data to inform robust estimates of false‐positive errors. As a result, it remains common practice to ignore or make unrealistic simplifying assumptions about false‐positive error rates when modeling species occurrence probabilities. However, this type of detection error can occur if surveyors misidentify a species via visual or auditory cues, or misidentify indirect sign (scat, tracks, and other species‐specific sign) as indicators of presence. False‐positive detection errors are known to occur in standard wildlife surveys (McClintock et al., 2010). For example, use of presence–absence data for 10 anuran species across the Northeastern United States revealed that when false‐positive error was not accounted for, occupancy estimates were biased by up to 70% for four of the 10 species studied (Ruiz‐Gutierrez et al., 2016). Other studies have documented similar biases resulting from failure to account for false‐positive errors (Clement et al., 2014; Royle & Link, 2006). As such, it is critical to model these potential detection errors.

For inconspicuous species, occupancy models tend to rely on indirect signs of presence. For example, Karanth et al. (2011) assessed the distribution of a population of tigers in India using indirect sign such as scat and tracks to inform occupancy models. It is increasingly common for researchers to infer occupancy status on the basis of tracks, scat, hair/fur middens, and even eDNA (Da Silva Neto et al., 2020; Nichols et al., 2008; Schmidt et al., 2013). In addition to these methods being low cost and noninvasive, it is important to use indirect sign where species may be missed if relying on direct sign alone. However, potential for quick decay of sign can add difficulty to assigning an occupied or unoccupied status at a given site and easily leads to higher risk of detection errors. On the other hand, long‐term preservation of sign can lead surveyors to mistakenly classify formerly occupied sites as currently occupied. Furthermore, misidentification of indirect sign by surveyors may result in either false‐positive or false‐negative errors. Finally, additional biases may be induced if environmental drivers differentially influence the reliability of sign types (e.g., precipitation or temperatures may affect scat decay rates), or if geographic regions differ in prevalence of reliable sign types. A “multi‐sign” occupancy modeling approach accounting for different detection rates and responses to environmental drivers across sign types may help reduce these sources of bias. Because both direct and indirect sign type detection can vary by region, accounting for each observation process separately as a function of a set of climatic and environmental covariates may allow for a more realistic representation of occupancy.

Nichols et al. (2008) was among the first to discuss the use of multiple sign types for fitting occupancy models. More recently, several studies have harnessed multiple detection methods to enable robust estimation of false detection rates (Guillera‐Arroita et al., 2017; Miller et al., 2011). Other studies, such as Clement et al. (2014), have also allowed for false detections, but have not estimated unique false detection rates across multiple sign types, nor have they investigated how environmental drivers differentially affect detection or false detection rates for each unique sign type. Furthermore, Chambert et al. (2015) and others (e.g., Kéry & Royle, 2021) have reinforced the importance of accounting for false detections and provided a framework and lexicon for building hierarchical false‐positive models. Our multi‐sign approach expands upon previous studies by modeling the detection process (both true and false detection probabilities) for each sign type as independent functions of unique covariate sets (e.g., environmental drivers). Our approach enables us to evaluate the shifting reliability of each sign type across space and time, and thereby help to develop more informed and robust survey protocols.

While this approach can be applied to any species for which surveys rely on multiple sign types as evidence of occupancy, we test this approach using American pika (*Ochotona princeps*), an inconspicuous species thought to be vulnerable to climate change across portions of its range due to potential upslope range shifts, subsequent vegetation availability, and sensitivity to extreme temperature changes (Beever et al., 2010; Erb et al., 2011, 2014; Jeffress et al., 2013). As such, occupancy models are critical to monitoring these potentially vulnerable populations and assessing the role of environmental and climatic factors as drivers of shifting occupancy patterns and range margins. Pika are an excellent test case for a multi‐sign occupancy modeling approach, as surveys for American pika typically monitor multiple indirect sign types—notably, scat and haypiles (piles of vegetation thought to serve as a key winter food resource; Dearing, 1997; Millar & Zwickel, 1972) in addition to direct observations of pika and auditory detections of the unique call produced by this species (Conner, 1985). These sign types differ in regional prevalence—for example, direct observations are common in some areas and rare in others—and are also likely to vary in reliability. Since scat and haypiles can persist in the environment for over 400 years (Castillo et al., 2016; Nichols, 2010), surveyors must be able to discriminate between fresh and old observations of indirect sign types in order to use these observations to infer current occupancy status. Fresh haypiles can be difficult to accurately distinguish from old haypiles, especially in lower elevations (Shinderman, 2015) where a few freshly browsed sprigs on an old pika haypile may be deposited by other sympatric herbivore species such as marmots or woodrats, raising the potential for false detections. Although previous evidence (Nichols, 2010) indicates fresh scat is easily distinguishable from old scat using color and consistency, anecdotal evidence suggests fresh scat decays quickly in the sun and especially at lower elevation sites and that determining the apparent age of scat may be regionally and even patch dependent (M. Jeffress and M. Shinderman, Personal communication, November 2021). Because scat is typically the most abundant sign detected in pika surveys, accurately assessing the age of scat detections and being able to distinguish fresh from old is crucial for generating precise and unbiased occupancy estimates.

In this study, we model occupancy dynamics for American pika using multiple direct and indirect indicators of pika presence (fresh scat, fresh haypiles, calls, and sightings) collected from 2010 to 2021 at five national parks in the Pacific Northwest. Furthermore, we investigate how estimates of pika occupancy trends and environmental drivers differ under four increasingly realistic representations of the pika observation process: (1) perfect detection (a common assumption for modeling pika occupancy), (2) standard occupancy model (single observation process with no possibility of false detection), (3) a multi‐sign occupancy model with no false positive detections (non‐false positive model), and (4) a multi‐sign occupancy model with false positive detections (full model). For the multi‐sign occupancy models, we modeled each observation process separately as a function of climatic and environmental covariates including substrate complexity, season, survey period, and vegetation cover. In addition, we modeled each occupancy process (initial patch occupancy, colonization, and extinction) separately as a function of covariates including temperature, precipitation, forb, rock, and shrub cover. Our primary objective was to model occupancy using both false‐positive and false‐negative detection probabilities for individual sign type. We also predicted that our “multi‐sign” approach for modeling observation error would alter estimates of occupancy processes (e.g., colonization, extinction) and its environmental drivers relative to more standard occupancy models in complex and unexpected ways. We also sought to model occupancy dynamics of pika across our study region as a function of climatic and environmental covariates and to assess how these estimates change under our four different treatments of observation error. We expected that accounting for false‐positive detection errors across sign types would reduce estimates of mean occupancy because failure to account for false detections typically results in upward bias in occupancy estimates (Royle, 2006). We anticipate that the results of this study will have broad implications for other systems in which multiple sign types are used to assess regional occupancy dynamics for other inconspicuous species.

## 2 METHODS

### 2.1 Study species

The American pika is a small lagomorph inhabiting patchy talus slopes across diverse ecosystems of the western US American pika do not hibernate and are generalist herbivores that often store vegetation caches called “haypiles” for winter food (Smith, 1974). Food caches serve as both winter diet and insulation in talus and are considered the major source of food for pika during winter (Dearing, 1997; Millar & Zwickel, 1972). American pika have long been considered to exhibit classical metapopulation dynamics (Hanski & Gilpin, 1991; Kreuzer & Huntly, 2003; Moilanen et al., 1998; Peacock & Smith, 1997). In 2010, American pika were considered for listing under threatened or endangered status by the United States Fish and Wildlife Service (Wolf et al., 2007), but were subsequently denied listing in 2010 due to both insufficient evidence of decline across known populations and a lack of survey data across much of their range. As such, considerable attention has been given to addressing how pika respond to climatic changes (Beever et al., 2003, 2011; Jeffress et al., 2013; Schwalm et al., 2016; Wolf et al., 2007) across their range. This species is a highly sensitive thermoregulator and may adjust their activity levels based on heat and cold exposure (MacArthur & Wang, 1973; Smith, 1974). While they are more commonly found above 2500 m, American pika metapopulations are also present in habitat below 2500 m (Ray et al., 2016; Rodhouse et al., 2010; Shinderman, 2015; Simpson, 2009). A wide range of factors, operating at different spatial scales, may promote or inhibit the persistence of pika populations including climate, substrate complexity, and available vegetation.

### 2.2 Occupancy survey and study area

**FIGURE 1:**
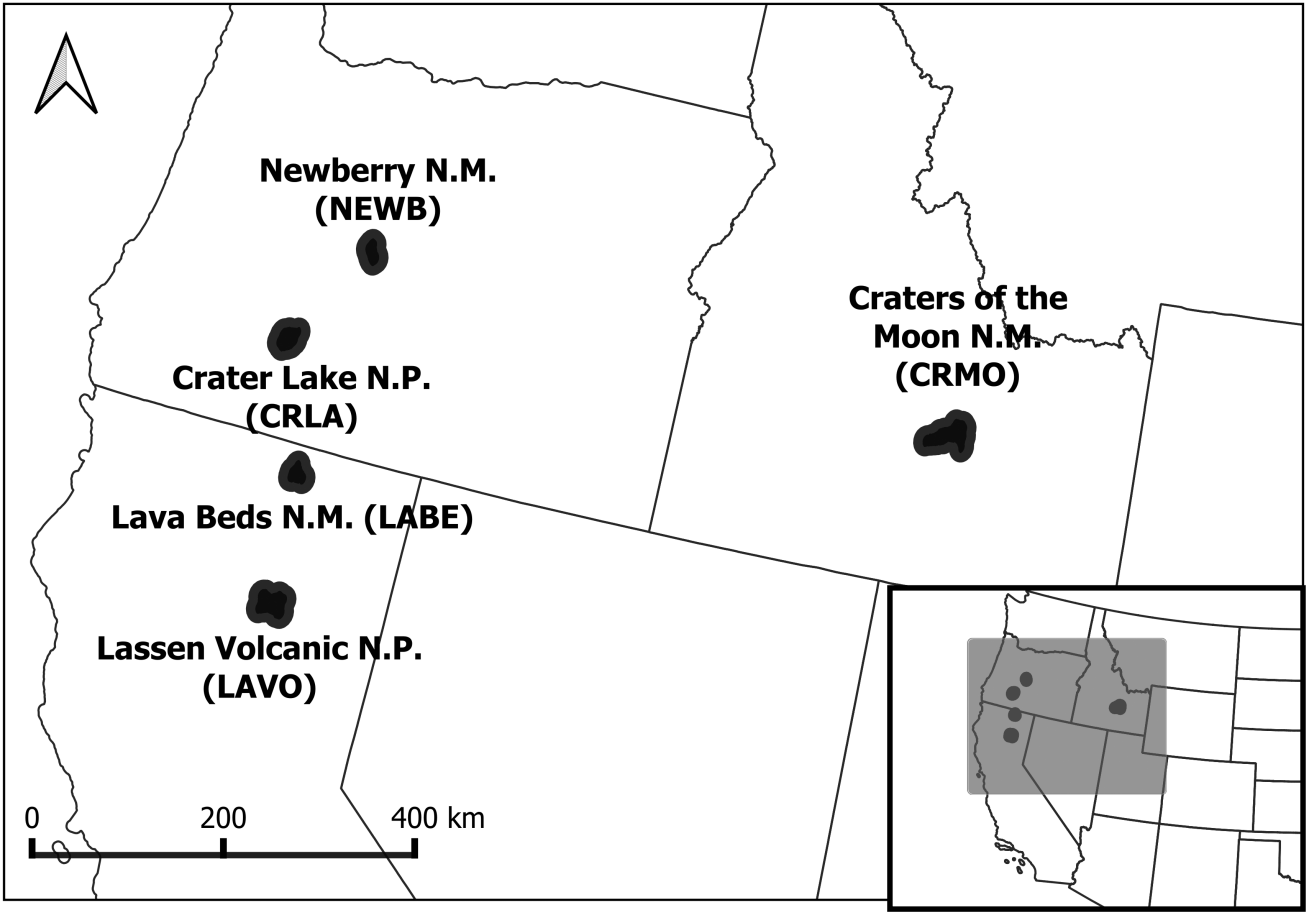
US Pacific Northwest map identifying 2010–2021 American pika survey areas within Newberry National Monument, Crater Lake National Park, Lava Beds National Monument, Lassen National Volcanic Park and Craters of the Moon National Monument.

We modeled American pika occupancy dynamics using two related datasets: The first is a 2010–2014 monitoring dataset established by the US National Park Service and the second a 2017–2021 dataset established by the Oregon State University Cascades HERS Lab with support from the National Park Service. Surveys occurred between 2010 and 2021 across five park units within the states of Oregon, Idaho, and California in the Pacific Northwest, including: Crater Lake National Park (CRLA), Craters of the Moon National Monument and Preserve (CRMO), Lassen Volcanic National Park (LAVO), Lava Beds National Monument (LABE), and Newberry National Monument (NEWB; Figure 1). Survey plots were designed to represent a typical pika territory size (Smith & Weston, 1990) and comprised a 12‐m radius circular surveyed area (following Jeffress et al. (2010); also used by Rodhouse et al. (2010) and Ray et al. (2012)). An average of 100 plots (hereafter referred to as “sites”) per park were surveyed each year. Resurveys were conducted for 30% of the sites within 1 month of original surveys each year to assess detection probability (Rodhouse et al., 2010). Surveyors spent up to 30 min searching a site and recorded up to 10 sign observations per survey (with 10 being considered a reasonable cap based on the density of sign typically observed within the sites and the time it takes to complete the survey). Observations comprised fresh or old scat (FS/OS), fresh or old haypiles (FH/OH), pika calls (PC), and pika sightings (PS). In practice, there were very few observations in any park with 10 or more instances of sign at a site within a single survey. Furthermore, given the amount of time spent in sites observing sign, it is very unlikely that any haypiles present would not have been detected if they were visible. The most likely scenario in those cases is that there either were no haypiles or they were not detectable (deep below the surface). For sites with multiple surveyors, independent blind surveys or double observer surveys that communicated results together for a combined “up to 30 min” were conducted. With two or more surveyors, surveys were not considered complete until both observers searched all large crevices and beneath large rocks. Surveyors ranged in experience level from newly trained to experienced. Only sign that observers felt they could confidently identify as pika were recorded. If a pika call was heard near the boundary of the site and not clearly within the site, it was determined to be outside the site. Observations of old sign were not included in our analyses.

**FIGURE 2:**
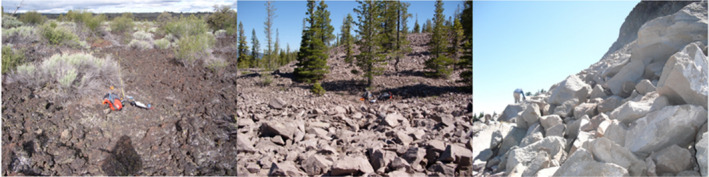
Example of low, intermediate, and high site‐level complexity in Lava Beds National Monument (1), Lassen Volcanic National Park (2), and Crater Lake National Park (3; Photos: OSU‐Cascades HERS Lab Unpublished Protocol Revision 3 Update, June 2019).

In addition to collecting records of pika sign, surveyors also classified each survey site according to three levels of rock (talus or lava) complexity: low, intermediate, and high (Figure 2). Lastly, surveyors used Daubenmire's (1959) method to estimate site‐level percent cover of rock, bare ground (dirt, mineral soil, and litter), forbs (non‐graminoid flowering herbaceous plants), grasses (graminoids [grasses and sedges]), shrubs (woody plants), and trees (Jeffress et al., 2010). A complete description of survey methods can be found in Jeffress et al. (2010) and Rodhouse et al. (2010).

To enable identifiability of false detection rates (we assume identifiability issues would arise if unambiguous detections were not available; Guillera‐Arroita et al. (2017)), we classified sign observations as ambiguous (potentially misidentified) and unambiguous (whereby a false detection was virtually impossible; Rodhouse et al., 2018). We used written notes recorded by surveyors in 2019–2021 surveys (during which note‐taking was consistent across all surveyors) to classify each sign observation as ambiguous or unambiguous. All fresh scat observations indicating the presence of fresh mucous holding scat together (*stuck, pyramid, tower, perched, stacked, teepee, cluster*) were designated as unambiguous, as such observations are necessarily fresh and are uniquely characteristic of pika scat. Fresh scat detections containing none of these keywords were considered ambiguous (some possibility of false detection i.e., mistaken for scat deposited by pika in a prior year before a site became extirpated). Fresh haypiles were also treated as an ambiguous sign type given the difficulty of knowing whether the vegetation was browse from another species (marmot or woodrat) and uncertainty around the quantity of browse that is to be considered a haypile. All pika sightings were treated as unambiguous. Pika have a distinctive size and shape relative to other small mammals in the study areas, and observers only recorded a pika sighting for high‐confidence observations; if an observer saw a small mammal in the site but could not confirm it was a pika, the observation was not recorded as a pika sighting. In addition, we designated as unambiguous all observations of fresh scat in which one or more fresh haypiles were observed during the same survey (M. Jeffress and M. Shinderman, Personal communication, November 2021).

### 2.3 Environmental and climatic covariates

Environmental covariates provided by the NPS that were considered in the model included survey season, site‐level cover class, and substrate complexity. A fourth covariate, “survey period” was used to distinguish between the 2010–2014 survey period and 2017–2021 period. These periods denote differences in maximum number of surveyors of a given survey. In the pre‐2017 period, surveys were conducted by one observer whereas in the post‐2017 period 2–4 observers conducted surveys; however, preliminary models suggested little to no effect of observer experience on detection outcomes. Thus, observer experience was not included in the final model. Climatic covariates included in the model, maximum mean summer temperature, and annual sum of daily precipitation for the previous water year were obtained from publicly available online sources (Table 1). Substrate complexity was centered to zero and all other non‐binary covariates were scaled for comparison across covariates.

**TABLE 1:** Occupancy and observation process parameters.

Occupancy process covariates		
Short name	Full name	Source
forb	Percent forb cover	NPS database
rock	Percent rock cover	NPS database
shrub	Percent shrub cover	NPS database
cplx^a^	Rock/substrate complexity	NPS database
tmaxmean	Mean daily max summer temp (°C)	gridMET (Abatzoglou, 2013)
precip	Sum daily precip over water yr (mm)	gridMET (Abatzoglou, 2013)
Observation process covariates		
Survey period	pre‐2017 or post‐2017 survey	NPS database
ssn	season (summer or fall)	NPS database

*Note*: NPS database data provided by National Park Service, all other covariate data taken from publicly sourced data sources.

^a^ Missing complexity data for Lava Beds National Monument (LABE).

### 2.4 Statistical analysis

#### 2.4.1 Selection of environmental covariates

Prior to fitting our dynamic occupancy model (see occupancy process model section), we determined which vegetation and climatic variables to use for modeling site‐level extinction and colonization processes using an initial model selection procedure (reverse step‐wise AIC; Akaike, 1974). We fitted a logistic regression model for the extinction process that included all vegetation cover and climatic variables, and which was fitted using data for all site‐year combinations that were occupied in the previous time‐step (i.e., observations exposed to potential extinction). For this preliminary modeling step, we assumed perfect detection and no false‐positive errors (i.e., surveys in which fresh sign was observed were presumed occupied). Similarly, we fitted a model for the colonization process that included all vegetation cover and climatic variables, using data for all site‐year combinations that were unoccupied in the previous time‐step (i.e., exposed to potential colonization). For each of these models, we then performed reverse step‐wise selection with AIC using the step function in R v4.0.1 (R Core Team, 2020) and incorporated the variables remaining in each model to fit the integrated Bayesian occupancy model (Appendix S2, Table 4). We chose covariates for the observation model based on a combination of previous studies demonstrating specific influences on pika occupancy (Erb et al., 2014; Huntly et al., 1986; Jeffress et al., 2013; Rodhouse et al., 2010) and ecological relevance (Appendix S2, Table 5). All quantitative covariates included in the Bayesian occupancy model were <75% correlated.

#### 2.4.2 Occupancy process model

Our process model followed a standard dynamic occupancy modeling framework with three distinct processes: (1) initial occupancy, (2) colonization of previously unoccupied patches, and (3) extinction of previously occupied patches (MacKenzie, 2002; Royle, 2004). Initial occupancy status $z_{i1}$ (where i is site) was modeled as a Bernoulli random variable, in which the probability of initial occupancy $\Lambda_{1}$ was a logit‐linear function of substrate complexity which was shown to influence pika occupancy in (Rodhouse et al., 2010). In subsequent years, the true occupancy status, $z_{\mathrm{it}}$ was modeled as a Bernoulli random variable with a conditional probability based upon prior‐year occupancy status $z_{i_{t-1}}$, using:

(1) $$z_{\mathrm{it}}∼\left\{\begin{array}{ll} \text{Bernoulli}\left(1-\varepsilon_{\mathrm{it}}\right), & \text{if}\;z_{i_{t-1}}=1\\ \text{Bernoulli}\left(\gamma_{\mathrm{it}}\right), & \text{if}\;z_{i_{t-1}}=0, \end{array}\right.$$

where $\gamma_{\mathrm{it}}$ is the probability of colonization in site i from year t−1 to year t, and $\varepsilon_{\mathrm{it}}$ is the probability of extinction in site i from year t−1 to year t. Probabilities of extinction and colonization were also modeled as logit‐linear functions of environmental covariates (Table 1). Random intercept terms on colonization and extinction were added to allow for variation among parks and year. A random slope term on initial occupancy was added to allow for among‐park variation in the effect of substrate complexity (fully derived equations can be found in Appendix S1).

#### 2.4.3 A “multi‐sign” model of the observation process

Each direct and indirect pika sign‐type: fresh scat (FS), fresh haypile (FH), pika call (PC), and pika sighting (PS), hereafter, “sign,” was treated as an independent detection process that reflects the underlying occupancy status of each site. For each sign type, observations were summarized by survey. We treated all survey‐level observations as binary (one or more observations of a sign type in a given survey was classified as 1, otherwise 0). We also constructed and fit multi‐sign models that treated FS and FH as count data (total number of observations recorded in each survey), but goodness‐of‐fit tests indicated poor fit and therefore we did not interpret these models further. Probability of detection for each sign type was modeled as a mixture process, allowing for true detections and false‐positive detections, following Royle and Link (2006). For each sign type, we modeled the survey‐specific probability of correct detection $p_{\mathrm{sign}}$ and false detection $p_{\mathrm{sign}}^{\xi }$ as a logit‐linear function of covariates:

(2) $$\text{logit}\left(p_{\mathrm{sign}}\right)=X\beta_{\mathrm{sign}},$$

(3) $$\left\{\begin{array}{ll} \text{logit}\left(p_{\mathrm{sign}}^{\xi }\right)=X_{p^{\xi }}\beta_{p^{\xi }}, & \text{if}\;\text{unambiguous}=0\\ p_{\mathrm{sign}}^{\xi }=0, & \text{if}\;\text{unambiguous}=1, \end{array}\right.$$

where $X\beta_{\mathrm{sign}}$ stands for a logistic regression of $p_{\mathrm{sign}}$ and $p_{\mathrm{sign}}^{\xi }$ for each site, year, and survey as a function of multiple covariates **X** (with regression coefficient vector $\beta_{\mathrm{sign}}$). Thus, if an observation is deemed “unambiguous,” the site is presumed occupied. We modeled the unconditional probability of detection for each sign type (${\mathrm{pd}}_{\mathrm{sign}}$—representing the probability of either false or correct detection of a given sign type) as conditional on the current occupancy status following:

(4) $${\mathrm{pd}}_{\mathrm{sign}}∼\left(\begin{array}{cc} p_{\mathrm{sign}}, & \text{if}\;z=1\\ p_{\mathrm{sign}}^{\xi }, & \text{if}\;z=0, \end{array}\right.$$

where the sign‐specific probability of detection ${\mathrm{pd}}_{\mathrm{sign}}$ for each site, year, and survey is equivalent to the probability of correct detection $p_{\mathrm{sign}}$ if a site is occupied and the probability of false detection $p_{\mathrm{sign}}^{\xi }$ if a site is unoccupied.

#### 2.4.4 Prior model

We used vague normal priors to define coefficients for all logit‐linear predictors for both occupancy and detection processes. We used vague gamma priors to define variance parameters (e.g., hyperpriors on random effect terms) and uniform distributions to initialize all probability parameters (e.g., initial occupancy probability, baseline colonization, and extinction probabilities; Appendix S1).

#### 2.4.5 Posterior predictive check

Model goodness‐of‐fit was based on posterior predictive checks using a Bayesian posterior predictive *p*‐value calculating the sum of squared error for fresh scat, fresh haypile, and pika call detections where values close to 0.5 indicate good model fit (Gelman, 2014). This allowed us to examine the degree to which the dispersion of the observed data matched the dispersion of simulated data. Bayesian *p*‐values close to 0 or 1 indicate poor model fit and demonstrate weak support for interpretation of parameter estimates.

#### 2.4.6 Bayesian model implementation

We ran models in a Bayesian framework using Markov Chain Monte Carlo (Hooten & Hobbs, 2015). We used the software JAGS (Plummer, 2003) which was called from R using the “jagsUI” wrapper (Kellner, 2021) for “rjags” (Plummer, 2021). Samples were drawn from posterior distributions using three MCMC chains, 100,000 iterations thinned by a factor of 10, and the first 50,000 iterations were discarded as a burn‐in period. Chains were checked for convergence, and if R‐hat was >1.1 for any parameter, the model was rerun (Brooks & Gelman, 1998). We checked model convergence using Gelman‐Rubin diagnostics and by examining trace plots of the MCMC chains. All models were run in R software. Our MCMC results indicated convergence (R‐hat <1.1) for all model parameters and for all three alternative representations of detection probability. Bayesian p‐values indicated adequate model fit for all three detection models and unique sign types (.42<p<.50; Kéry, 2010).

#### 2.4.7 Comparing alternative observation models

To test the extent to which our most realistic and sophisticated model influenced the observation process relative to more conventional models, we ran three additional models with increasingly simple representations of the observation process: (1) non‐false‐positive model: a “multi‐sign” detection model with no potential for false detections (all direct or indirect pika observations imply that a site was currently occupied in a given year), (2) standard occupancy model: a model with a single observation process and no possibility of false detection, and (3) perfect detection model: a model in which all pika detection events were assumed to accurately reflect the underlying occupancy status and all surveys yielding no pika detections were assumed to reflect pika absence. In general, high detectability has been assumed for pika and thus detection error has generally been ignored in this modeling framework (Jeffress et al., 2013; Ray et al., 2016; Rodhouse et al., 2010). We computed site‐occupancy rates for each park and year by dividing the estimated number of occupied sites in each park (determined by monitoring the binary site‐occupancy status for each MCMC iteration) by the total number of monitored sites at each park. We then summarized occupancy rates for each park and year as a posterior mean (point estimate) and a 90% credible interval. We then ran weighted linear regressions of mean park‐level occupancy as a function of year (one regression model per park), using an inverse‐variance weighting technique (weights equal to the inverse of posterior variance of each site‐occupancy estimate), and interpreted significant trends at alpha = 0.1.

## 3 RESULTS

Overall, our survey dataset included a total of 5126 surveys of 771 unique sites across 5 parks and 12 years (Table 2).

**TABLE 2:** Park surveys by year.

	2010	2011	2012	2013	2014	2017	2018	2019	2020	2021
Original surveys across park										
CRLA^a^	85	108	141	101	102	0	95	100	0	96
CRMO^b^	56	135	146	103	103	103	99	96	100	100
LABE^c^	101	101	100	100	100	99	99	100	89	50
LAVO^d^	76	113	75	103	103	101	100	98	49	0
NEWB^e^	0	0	0	0	0	100	102	102	0	100
Resurveys across parks										
CRLA	50	30	30	41	30	0	29	29	0	0
CRMO	50	35	30	20	29	16	30	29	30	30
LABE	50	0	30	14	50	27	31	30	0	18
LAVO	41	30	0	21	20	27	29	29	0	0
NEWB	0	0	0	0	0	30	31	31	0	30

^a^ Crater Lake National Park.

^b^ Craters of the Moon National Monument and Preserve.

^c^ Lava Beds National Monument.

^d^ Lassen Volcanic National Park.

^e^ Newberry National Monument: surveys began in 2017.

Of these surveys, at least 30% were resurveys conducted within 2 weeks of the original survey to ensure no occupancy turnover. Mean maximum summer temperature (June–August) at our study sites ranged between 16.44 and 30.17°C. The sum of annual precipitation ranged from 101 to 5130 mm (Appendix S2, Figure 5).

### 3.1 Pika occupancy dynamics

**FIGURE 3:**
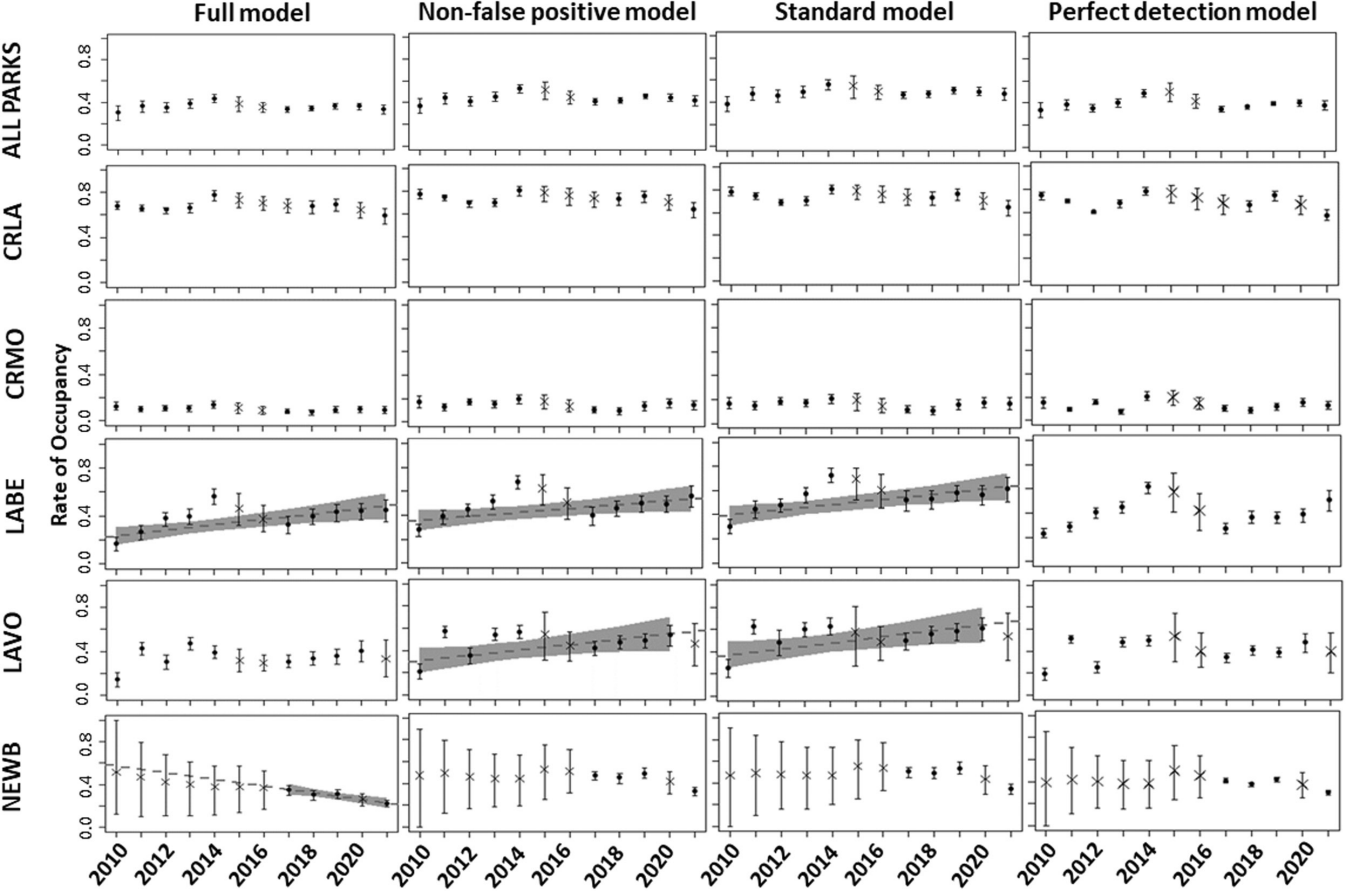
Comparison of posterior site‐occupancy rates (filled circles represent posterior means, error bars represent 90% credible intervals) through time for all parks across all models. Shaded regions indicate post hoc linear regression models with a 90% C.I. (using inverse‐variance weighting to account for uncertainty of point estimates). Only those regression relationships significant at alpha = 0.1 are depicted. Points represented with an “X” indicate years that the park was not surveyed.

The colonization rate was 5.8%, and extinction rate was 6.2%. Across our study period, we detected two significant park‐level trends: occupancy increased from 19% to 40% at LABE and decreased from 40% to 22% in NEWB. There was no detectable trend in occupancy across all parks and occupancy ranged from 32 to 40% (Figure 3; Appendix S2, Table 6 and 7).

Rock complexity was positively correlated with initial occupancy ($\alpha_{\Lambda_{\text{complexity}}}=1.325$, 95% CRI: 0.773 to 1.944, $\omega^{+}=1.0$; where $\omega^{-}$ or where $\omega^{+}$ indicate weight of evidence above or below zero and point estimates represent the posterior mean). Percent forb cover was negatively associated with the probability of extinction ($\alpha_{\varepsilon_{\text{forb}}}=-2.700$, 95% CRI: −4.458 to −1.265, $\omega^{-}=0.988$) and weakly associated with probability of colonization ($\alpha_{\gamma_{\text{forb}}}=0.033$, 95% CRI: −0.303 to 0.304, $\omega^{+}=0.877$). Shrub cover ($\alpha_{\gamma_{\text{shrub}}}=0.255$, 95% CRI: 0.044 to 0.470, $\omega^{+}=0.989$) was positively correlated with colonization (Appendix S1, Table 7).

Correlation in among‐year process variation (colonization and extinction processes) between parks varied from 0.63 to −0.43 (Appendix S2, Table 8). The highest positive correlations (0.63 for colonization, 0.61 for extinction) were observed between the two parks with highest average temperatures and lowest average precipitation (LABE and CRMO). Other parks with positively correlated annual colonization rates included CRMO and CRLA (0.564), CRLA and LAVO (0.561), and CRLA and NEWB (0.575; Appendix S2, Table 8).

### 3.2 Observation process

Total detection probability across sign types was 82%. Overall sign detection rates, individual sign type detection rate, and associated false‐positive error rates varied among parks. The average probability of correct detection in CRLA was the highest at 92.4% and lowest in NEWB at 77.1% (Table 3).

**TABLE 3:** Detection rates^a^ across park and sign type fresh scat (FS), fresh haypiles (FH), pika calls (PC), and pika sightings (PS): full model.

	True detection rate					False‐positive detection rate			
Park	Overall	FS	FH	PC	PS	Overall	FS	FH	PC
CRLA	0.924	0.806	0.309	0.599	0.194	0.110	0.017	0.020	0.076
CRMO	0.785	0.722	0.366	0.091	0.016	0.028	0.007	0.017	0.004
LABE	0.764	0.719	0.150	0.014	0.004	0.065	0.045	0.015	0.005
LAVO	0.859	0.626	0.404	0.345	0.060	0.108	0.018	0.049	0.043
NEWB	0.771	0.540	0.144	0.301	0.106	0.117	0.016	0.016	0.086
AVG	0.820	0.683	0.275	0.270	0.076	0.086	0.020	0.023	0.043

^a^ Using the raw data of sign type observations for each site/year combination, the probability of either true or false detection was determined conditional on the site being occupied or unoccupied (per the posterior distribution for the binary occupancy variable at each unique site and year). Probabilities were computed as weighted averages across surveys.

Rate of fresh scat detections ($p_{\mathrm{fs}}=0.801$, 95% CRI: 0.764 to 0.801) was substantially higher than fresh haypiles ($p_{\mathrm{fh}}=0.288$, 95% CRI: 0.248 to 0.331). Mean detection rate for pika calls ($p_{\mathrm{pc}}=0.412$, 95% CRI: 0.375 to 0.451) was substantially higher than pika sightings ($p_{\mathrm{ps}}=0.134$, 95% CRI: 0.110 to 0.160). Survey period was negatively associated with number of fresh scat detections ($\beta_{{\mathrm{FS}}_{\text{survey}\;\text{period}}}=-0.684$, 95% CRI: −0.927 to −0.442, $\omega^{-}=1.0$). Seasonality (summer vs. fall) affected the probability of detecting pika calls ($\beta_{{\mathrm{PC}}_{\text{season}}}=-0.544$, 95% CRI: −0.749 to −0.344, $\omega^{-}=1.0$) and sightings ($\beta_{{\mathrm{PS}}_{\text{season}}}=-0.584$, 95% CRI: −0.906 to −0.273, $\omega^{-}=1.0$). Sites with higher substrate complexity tended to have fewer detectable fresh haypile detections ($\beta_{{\mathrm{FH}}_{\text{complexity}}}=-0.262$, 95% CRI: −0.494 to −0.025, $\omega^{-}=1.0$), pika calls ($\beta_{{\mathrm{PC}}_{\text{complexity}}}=-0.273$, 95% CRI: −0.476 to −0.072, $\omega^{-}=1.0$), and pika sightings ($\beta_{{\mathrm{PS}}_{\text{complexity}}}=-0.306$, 95% CRI: −0.583 to −0.030, $\omega^{-}=0.985$). Lastly, higher forb cover was associated with a higher probability of detecting haypiles ($\beta_{{\mathrm{FH}}_{\text{forb}}}=0.336$, 95% CRI: 0.239 to 0.438, $\omega^{+}=1.0$; Appendix S2, Figures 8–11; Table 7).

The average overall false‐positive rate was 8.6%. The average false‐positive rate was highest in NEWB (0.117) and lowest in CRMO (0.028; Table 3). The highest false‐positive error rates were associated with pika call ($p_{\mathrm{pc}}^{\xi }=0.042$, 95% CRI: 0.031 to 0.055) followed by fresh scat ($p_{fs}^{\xi }=0.037$, 95% CRI: 0.020 to 0.055). The mean false‐positive rate for fresh haypiles was extremely low ($p_{\mathrm{fh}}^{\xi }=0.020$, 95% CRI: 0.012 to 0.031). False‐positive scat detections tended to be higher during the 2017–2021 surveys versus the 2010–2014 survey period ($\beta_{{\mathrm{FS}}_{\text{season}}}^{\xi }=-1.139$, 95% CRI: −2.077 to −0.358, $\omega^{-}=1.0$). False pika call detections were more likely in summer than in fall ($\beta_{{\mathrm{PC}}_{\text{season}}}^{\xi }=-1.069$, 95% CRI: −1.678 to −0.529, $\omega^{-}=1.0$). Higher substrate complexity was associated with fewer false pika call detections ($\beta_{{\mathrm{PC}}_{\text{complexity}}}^{\xi }=-0.228$, 95% CRI: −0.640 to 0.193, $\omega^{+}=0.974$). Lastly, false‐positive fresh haypile detections were more common in areas with higher forb cover ($\beta_{{\mathrm{FH}}_{\text{forb}}}^{\xi }=0.333$, 95% CRI: 0.143 to 0.503, $\omega^{+}1.0$; Appendix S2, Table 7).

### 3.3 Comparing alternative observation models

Temporal (year to year) variance across parks for mean rate of extinction tended to be greatest in the full model ($\mu_{\sigma }^{\varepsilon }=0.65$) and lowest in the perfect detection model ($\mu_{\sigma }^{\varepsilon }=0.40$), whereas variance across park for colonization was highest in the non‐false‐positive model ($\mu_{\sigma }^{\gamma }=0.65$) and lowest in the perfect detection model ($\mu_{\sigma }^{\gamma }=0.38$; Appendix S2). Variance across park for initial occupancy was similar in the full ($\mu_{\sigma }^{\Lambda }=1.13$) and perfect detection model ($\mu_{\sigma }^{\Lambda }=1.14$) and lowest in the non‐false‐positive model ($\mu_{\sigma }^{\Lambda }=0.91$). The estimate for initial occupancy in the full model was 28.4%, compared with 37.0% in the non‐false positive model, 39.5% in the standard detection model and 33.2% in the perfect detection model. Mean colonization rate was lowest in the full model (8%) and highest in the perfect detection model (16%). Mean extinction rate was lowest in the full model (6.2%) and highest in the perfect detection model (26.4%; Appendix S2, Table 7, 11, 13, 14).

Percent forb cover in the non‐false‐positive model indicated an increased negative effect on extinction ($\alpha_{\varepsilon_{\text{forb}}}=-1.948$, CRI: −3.098 to −1.065, $\omega^{-}=1.0$) and (shrub cover; Figure 6 and summarized in Appendix S2, Table 14). A notable difference in the perfect detection model included producing the weakest negative effects of forb cover on extinction ($\alpha_{\varepsilon_{\text{forb}}}=-0.420$, CRI: −0.679 to −0.185 $\omega^{-}=1.0$; Appendix S2, Table 14). Compared with the full model, the standard deviation of fraction of occupied sites across all parks was higher ($\sigma^{2}=0.0527$) than the full model ($\sigma^{2}=0.0351$).

## 4 DISCUSSION

The processes regulating the reliability and detectability of direct and indirect sign types are likely to vary across space and time even when identical protocols are applied to target organisms. When occupancy status is determined on the basis of multiple sign types, these differences could result in biased estimates of occupancy and its environmental drivers. A multi‐sign occupancy modeling approach like the one we present in this study allows unique detection processes to be modeled separately in order to more effectively model occupancy dynamics (MacKenzie et al., 2003). We found that distinct sign types exhibited substantial differences in overall reliability, false positive rates, and influence of environmental covariates.

Although occupancy models involving surveys of direct and indirect sign types typically ignore this variation—instead, treating all sign types equally in a pooled observation process—this practice may introduce previously unexplored sources of bias in occupancy estimation if different sign types vary in prevalence across sites and regions. We found that overall survey reliability varied substantially among the parks included in our study despite survey protocols being standardized across all parks, with mean true detection rates varying from 77.1% to 92.4% and mean false detection rates varying from 2.8% to 11.7% for different parks. This variation in survey reliability was presumably driven by differences in the reliability and prevalence of different sign types in each park (e.g., haypiles; see below). For example, detection rates of pika calls by park varied from 1.4% to 59.9%, and pika sightings varied from 0.4% to 19.4%. By modeling each distinct sign type separately, our “multi‐sign” occupancy models are able to accommodate substantial regional differences in survey reliability while producing unbiased estimates of occupancy processes. For instance, in the American pika system, haypiling may be particularly sensitive to environmental gradients. If not modeled explicitly this could cloud estimates of occupancy trends.

**FIGURE 4:**
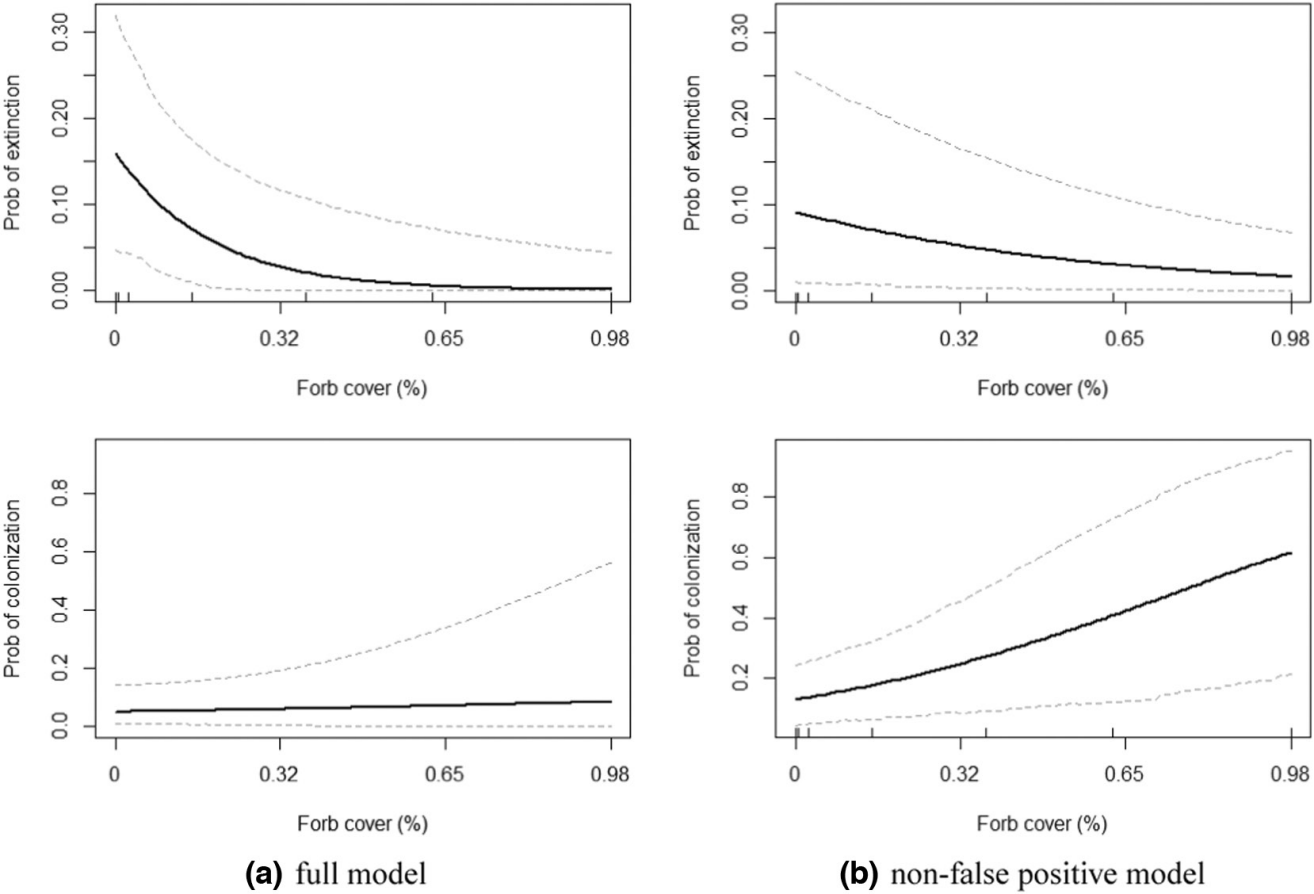
Effects plots comparing the relationships between occupancy processes (colonization and extinction probabilities) and forb cover for (a, left) a multi‐sign occupancy model with false detections and (b, right) an otherwise‐equivalent model with false detection rates assumed to be zero. Dashed lines indicate 95% Bayesian credible interval.

While false detections are ignored under most standard occupancy modeling frameworks (MacKenzie, 2002), researchers are increasingly allowing for false‐positive errors within occupancy modeling frameworks and acknowledging the important biases that can result from ignoring false‐positive errors (Berigan et al., 2019; Chambert et al., 2015; Clement et al., 2014; Miller et al., 2013; Royle & Link, 2006). Our study echoes these findings, suggesting that false detection rates can have strong effects on key inferences about occupancy and occupancy dynamics, including mean occupancy rates, the magnitude and significance of occupancy trends, and the environmental drivers of occupancy. Estimates of several key environmental drivers were also sensitive to changes in how the observation process was represented. For example, the strength of the estimated effect of substrate complexity on initial occupancy probability was much stronger (nearly double in magnitude) in the full model versus the perfect detection model. Finally, the estimated effect of forb cover on patch‐level extinction risk was also far stronger in the full model versus the perfect detection model; whereas the full model suggests that forb cover ≥ca. 65% implies a nearly 100% probability of site persistence, the perfect detection model implies that forb cover barely influences the probability of extinction (Figure 4). Overall, these results suggest that estimates of occupancy processes are sensitive to how sign types vary in reliability across geographic and environmental space, and failure to account for this variation may lead to biased or incomplete estimates of key occupancy processes. For example, forb cover more strongly influenced occupancy in the full model than in the non‐false positive, standard, and perfect detection models (Appendix S2, Table 7–14). Multi‐sign occupancy models should yield a more realistic understanding of occupancy dynamics in cases where detection processes differ in reliability and response to environmental gradients and vary in prevalence across study sites or regions.

Most previous occupancy models for pika and other inconspicuous species have ignored false detections based on the assumption of low false detection rates. In our model, misidentification of pika calls was surprisingly prevalent, averaging 4.3% and varying substantially among our study sites (0.5% to 8.6%). When ignoring the potential for false detections, mean occupancy rates increased by 9% across the study region relative to the model that accommodated false detection errors, most likely reflecting the upward biases induced by treating false detections as true. In contrast to our assumption that false detections would be driven primarily by indirect sign observations, one of the largest sources of false positive errors in our study was direct observation of pika calls. Coupled with the knowledge that pika calls were relatively uncommon relative to other sign types (27%, compared to fresh scat at 68.3%), this suggests that future survey protocols may wish to focus on other sign types as primary indicators of occupancy. On the other hand, the most likely explanation for the high false detection rate for pika calls is that surveyors correctly detected a pika but that the call emerged from outside the survey site; pika calls are unlikely to be confused with calls made by any other species. Therefore, ignoring false detections of pika calls may not pose a severe issue for occupancy estimation if researchers are more interested in estimating occupancy beyond the level of the standard survey site. Among the indirect sign types, fresh haypiles were associated with the highest rate of false detections (2.3%). These false detections are likely the result of mistaking old haypiles for fresh, and such errors are likely to induce upward biases in estimates of regional occupancy if not modeled explicitly. Rigorous simulation studies are needed to determine thresholds for both true and false detection rates beyond which these errors can be safely ignored. In the meantime, we suggest that researchers should incorporate both false‐negative and false‐positive detection errors in occupancy models unless strong evidence suggests that one or both of these errors can be ignored.

At the site level, detection probabilities for this species are generally thought to be high (≥0.9) across their range (Beever et al., 2008, 2010; Ray et al., 2012), leading many pika researchers to disregard detection errors as inconsequential sources of bias for estimating pika occupancy and metapopulation dynamics (Erb et al., 2011; Jeffress et al., 2013; Ray et al., 2016; Rodhouse et al., 2010; Shinderman, 2015). This assumption has been justified on the basis of previous findings that pika are detectable at a rate of ≥90% across their range (Beever et al., 2008, 2011; Jeffress et al., 2013; Ray et al., 2016; Rodhouse et al., 2010; Wilkening & Ray, 2016). Rodhouse et al., 2010 is the only one of these studies to actually estimate detection probability with replicate survey data. More recent research has suggested that the assumption of perfect detection may lead to biased results in occupancy and metapopulation models for this species due to imperfect detection as well as occasional false detections (Rodhouse et al., 2018). While our results confirm that the detection rate is high (82% true detection rate) for pika surveys, our results also suggest that failing to account for imperfect detection for pika in our study region can lead to biased estimates of occupancy trends and covariate effects and can result in substantially larger inter‐annual variance relative to models that account for imperfect detection. Given our results, we caution against ignoring observation errors and suggest that it is critical to model the observation process when study goals involve estimating occupancy dynamics and its environmental drivers, even for species with detection rates approaching or exceeding 90%.

In our “multi‐sign” occupancy modeling framework, we assumed that all unique sign types represented independent indicators of the underlying occupancy status. This implies that the probability of failing to detect all unique sign types during a single survey (probability of false negative) could be computed as the product of the probability of failing to detect each individual sign type, and that the probability of a false detection could be computed as the inverse of the product of correctly failing to observe each individual sign type. However, interactions among sign types are possible, and even likely; for example, two or more unique sign types may be extremely reliable indicators of occupancy when they co‐occur, whereas neither is reliable separately. In our study system, surveys in which both fresh scat and haypiles co‐occur were considered to be an unambiguous indicator of occupancy—which we modeled by disallowing false detections for such surveys. However, future multi‐sign occupancy frameworks should consider modeling interactions among sign types explicitly. Such models may more realistically describe how information from surveys with multiple distinct sign types can be used to most precisely describe the underlying observation process.

Failure to allow for detection errors that can and do occur in real‐world ecological surveys, such as false detections and use of sign types that vary in reliability, can lead to biased or incomplete estimates of key occupancy processes. Building on other occupancy models that handle false detections (Clement et al., 2014; Rodhouse et al., 2018), and multiple unique detection methods (Guillera‐Arroita et al., 2017; Kéry & Royle, 2021; Miller et al., 2011), we present a flexible approach for modeling detection probability for multiple sign types, whereby detection probability can respond to spatio‐temporal environmental gradients uniquely for each sign type. Unlike previous studies, our method also allows any sign type to be present in both ambiguous and unambiguous forms, enabling additional power to estimate false‐positive detection rates (Guillera‐Arroita et al., 2017). In theory, knowledge of the degree of ambiguity of different sign types could be used to improve survey efficiency by (e.g.) resurveying sites only until an unambiguous sign is observed (ceasing surveys at that point), allowing more effort to be put into other sites. However, in the context of monitoring pika populations, this might not save that much time, as multiple sign types, when present, can typically be observed and recorded quickly.

We anticipate that our method has broad applicability for other systems in which multiple sign types are used to assess regional occupancy dynamics, especially for inconspicuous species. As hierarchical models (often fitted in a Bayesian framework) become increasingly accessible to ecologists, incorporation of more realistic and complex models of the observation process, such as multi‐sign occupancy models, have become relatively straightforward to implement. We believe researchers can build upon our framework to develop more precise detection models that ultimately improve our ability to estimate regional occupancy and to develop more effective regional monitoring protocols. Ultimately, incorporation of multi‐sign detection probabilities in occupancy models has great potential to positively impact management and conservation decisions for inconspicuous species.

## AUTHOR CONTRIBUTIONS

**Mia R. Goldman:** Conceptualization (equal); formal analysis (lead); visualization (lead); writing – original draft (lead); writing – review and editing (equal). **Matthew Shinderman:** Conceptualization (supporting); data curation (lead); investigation (supporting); methodology (supporting); resources (supporting); writing – review and editing (supporting). **Mackenzie R. Jeffress:** Conceptualization (supporting); data curation (supporting); methodology (supporting); writing – review and editing (supporting). **Thomas J. Rodhouse:** Conceptualization (supporting); data curation (supporting); methodology (supporting); resources (supporting); writing – review and editing (supporting). **Kevin T. Shoemaker:** Conceptualization (equal); formal analysis (supporting); investigation (supporting); methodology (supporting); supervision (lead); validation (supporting); visualization (supporting); writing – original draft (supporting); writing – review and editing (supporting).

## Supporting information


Appendix S1
Appendix S2

## Data Availability

All data were collected by the OSU‐Cascades HERS Lab with support from the National Park Service under Cooperative Agreement P18AC01062. Use of these data should include an attribution of source to the OSU Cascades Human and Ecosystem Resilience and Sustainability Lab. Dryad repository: https://doi.org/10.5061/dryad.ttdz08m2n.
