# Supplementary material for: Integrating multiple sign types to improve occupancy estimation for inconspicuous species
Source: Ecol Evol. 2023 May 15;13(5):e10019. doi: 10.1002/ece3.10019 (PMC10183821; doi:10.1002/ece3.10019)
Supplement: Supplementary file 1 — Appendix S1 Appendix S2 [file ECE3-13-e10019-s001.pdf]

Appendix 1

Data model

$$y_{iv} \sim \begin{cases} Bernoulli(p), & \text{if } z_i = 1 \\ Bernoulli(p^{\xi}), & \text{if } z_i = 0. \end{cases} \tag{5}$$

Process model

$$z_{it} \sim \begin{cases} Bernoulli(1 - \varepsilon_{it}), & \text{if } z_{it-1} = 1 \\ Bernoulli(\gamma_{it}), & \text{if } z_{it-1} = 0 \end{cases} \tag{6}$$

$$\psi_t = \psi_{t-1}(1 - \varepsilon_{t-1}) + (1 - \psi_{t-1})(\gamma_{t-1}) \tag{7}$$

$$\gamma_t = \frac{\psi_{t+1} - \psi_t(1 - \varepsilon_t)}{1 - \psi_t} \tag{8}$$

$$\varepsilon_t = 1 - \frac{\psi_{t+1} - (1 - \psi_t)\gamma_t}{\psi_t} \tag{9}$$

$$\begin{aligned} logit(\varepsilon_{it}) &= \alpha_0^{\varepsilon} + \alpha_1^{\varepsilon} X_{it} + \alpha_2^{\varepsilon} X_{it} + \alpha_3^{\varepsilon} X_{it} + \eta_r + Y_{rt} \\ logit(\gamma_{it}) &= \alpha_0^{\gamma} + \alpha_1^{\gamma} X_{it} + \alpha_2^{\gamma} X_{it} + \alpha_3^{\gamma} X_{it} + \alpha_4^{\gamma} X_{it} + \eta_r + Y_{rt} \\ logit(\psi_{i,t=1}) &= \alpha_0^{\psi} + \alpha_1^{\psi} X_i + \upsilon_i X_i + \eta_r \end{aligned} \tag{10}$$

Observation model

$$\begin{cases} logit(p^{\xi}_{sign}) = \mathbf{X}_{p^{\xi}}\boldsymbol{\beta}_{p^{\xi}}, & \text{if } unambiguous = 0 \\ p^{\xi}_{sign} = 0, & \text{if } unambiguous = 1, \end{cases} \tag{11}$$

$$logit(p_{sign_{it}}) \sim \begin{cases} \text{PS=}\beta_0 + \beta_{1(season_{itv})} + \beta_{2(complexity_i)} \\ \text{PC=}\beta_0 + \beta_{1(season_{itv})} + \beta_{2(complexity_i)} \\ \text{FS=}\beta_0 + \beta_{1(season_{itv})} + \beta_{2(surveyperiod_{itv})} + \beta_{3(complexity_i)} \\ \text{FH=}\beta_0 + \beta_{1(season_{itv})} + \beta_{2(surveyperiod_{itv})} + \beta_{3(forb_{it})} + \beta_{4(complexity_i)} \end{cases} \tag{12}$$

$$logit(p^{\xi}_{sign_{it}}) \sim \begin{cases} \text{PC=}\beta_0 + \beta_{1(season_{itv})} + \beta_{2(complexity_i)} \\ \text{FS=}\beta_0 + \beta_{1(season_{itv})} + \beta_{2(surveyperiod_{itv})} + \beta_{3(complexity_i)} \\ \text{FH=}\beta_0 + \beta_{1(season_{itv})} + \beta_{2(surveyperiod_{itv})} + \beta_{3(forb_{it})} + \beta_{4(complexity_i)} \end{cases} \tag{13}$$

$$pd_{sign_{it}} \sim \begin{cases} p_{sign_{it}}, & \text{if } z_{it} = 1 \\ p^{\xi}_{sign_{it}}, & \text{if } z_{it} = 0, \end{cases} \tag{14}$$

$$pd_{it} = 1 - (1 - pd_{FS}) * (1 - pd_{FH}) * (1 - pd_{PC}) * (1 - pd_{PS}) \tag{15}$$

Parameter model

Base occupancy processes were modeled using $Beta$(1,1) priors:

$$\begin{aligned} \psi_{(t=1)} &\sim Beta(1,1) \\ \gamma &\sim Beta(1,1) \\ \varepsilon &\sim Beta(1,1) \end{aligned} \tag{16}$$

Sign type probability of detection priors modeled as log-transformed beta distributions:

$$\begin{aligned} p_{fs} &\sim Beta(1,1) \\ p^{\xi}_{fs} &\sim Beta(1,1) \\ p_{fh} &\sim Beta(1,1) \\ p^{\xi}_{fh} &\sim Beta(1,1) \\ p_{pc} &\sim Beta(1,1) \\ p^{\xi}_{pc} &\sim Beta(1,1) \\ p_{ps} &\sim Beta(1,1) \end{aligned} \tag{17}$$

Environmental covariate priors modeled as normal distributions:

$$\begin{aligned} \boldsymbol{\alpha} &\sim N(\mathbf{0}, \sigma^2_{\alpha}\boldsymbol{I}) \\ \boldsymbol{\beta} &\sim N(\mathbf{0}, \sigma^2_{\beta}\boldsymbol{I}) \\ \sigma^2_{\alpha} &= 100 \\ \sigma^2_{\beta} &= 100 \end{aligned} \tag{18}$$

Hyperparameters[1]:

$$
\begin{aligned}
\eta_r &\sim N(0, \sigma^2_\eta) \\
Y_{rt} &\sim N(0, \sigma^2_\eta) \\
\sigma_\eta &\sim Gamma(1, 1) \\
\upsilon_i &\sim N(0, \sigma^2_\upsilon) \\
\sigma_\upsilon &\sim Gamma(0.1, 0.1)
\end{aligned}
\tag{19}
$$

[1] η_r and Y_{rt} indicate random intercepts for initial occupancy, colonization, and extinction models represented in JAGS as raw probabilities (e.g. not on logit scale).

Appendix 2

Table 4: Table of covariates for probability of initial occupancy, extinction and colonization (X* = included in final Bayesian models X = considered but not included in the final model)

variable	initial occupancy	extinction	colonization
percent forb cover		X*	X*
percent rock cover		X	X*
percent shrub cover		X	X*
rock complexity	X*	X	X
sum daily water year precipitation		X*	X
average water year snow pack		X	X
mean maximum summer temperature		X*	X*

Table 5: Table of covariates for American pika observation processes where the probability of detection is modeled separately for individual sign types [a]. (X* = included in final Bayesian models)

covariate	Indirect sign				Direct sign		
	FS[b] ***C***	FS ***F***	FH ***C***	FH ***F***	PC ***C***	PC ***F***	PS[c] ***C***
substrate complexity	X*	X*	X*	X*	X*	X*	X*
season	X*	X*	X*	X*	X*	X*	X*
survey period	X*	X*	X*	X*			
% forb cover			X*	X*			
observer experience	X	X	X	X			

[a]FS: Fresh scat, FH: fresh haypiles, PC: Pika call, PS: pika sighting

[b]Some sign types [FS, FH] were associated with both a probability of correct detection (C) and a probability of false detection (F)

[c]PS was assumed to be the only sign type without ambiguity

Table 6: Description of Greek symbols for model terms

Greek	Parameter term
Ξ	base occupancy rate
ψ	probability of occupancy
γ	probability of colonization
ε	probability of extinction
Λ	initial occupancy
η_r	random effect of region
Y_{rt}	random effect of region within year
υ_i	random effect of complexity on initial occupancy
α	covariate effects on occupancy processes
β	covariate effects on observation processes
X	covariate matrix
μ_{sign}	mean sign type detection rate
ξ	false positive detection error

Table 7: 95% Credible intervals, standard deviation and mean of posterior estimates: full model

Parameter	mean	sd	2.5%	50%	97.5%
**overall occupancy dynamics/detection rates**					
Ω_Ξ	0.284	0.156	0.070	0.253	0.667
γ_Ξ	0.058	0.059	0.015	0.043	0.212
ε_Ξ	0.062	0.056	0.009	0.046	0.225
p_{fs}	0.801	0.019	0.764	0.801	0.837
p^{ξ}_{fs}	0.036	0.009	0.020	0.035	0.055
p_{fh}	0.288	0.021	0.248	0.288	0.331
p^{ξ}_{fh}	0.020	0.005	0.012	0.020	0.031
p_{pc}	0.412	0.019	0.375	0.412	0.451
p^{ξ}_{pc}	0.042	0.006	0.031	0.042	0.055
p_{ps}	0.134	0.013	0.110	0.133	0.160
**occupancy process parameter estimates**					
$\alpha_{\Lambda_{complexity}}$	1.325	0.297	0.773	1.313	1.944
$\alpha_{\varepsilon_{forb}}$	-2.700	0.824	-4.458	-2.638	-1.265
$\alpha_{\varepsilon_{temp}}$	-0.078	0.703	-1.268	-0.140	1.407
$\alpha_{\varepsilon_{ppt}}$	-1.082	0.618	-2.363	-1.058	0.039
$\alpha_{\gamma_{forb}}$	0.033	0.153	-0.303	0.043	0.304
$\alpha_{\gamma_{shrub}}$	0.255	0.108	0.044	0.255	0.470
$\alpha_{\gamma_{rock}}$	0.259	0.153	-0.029	0.256	0.567
$\alpha_{\gamma_{temp}}$	-0.252	0.273	-0.789	-0.251	0.281
**observation process parameter estimates**					
$\beta_{FS_{survey\ period}}$	-0.684	0.124	-0.927	-0.685	-0.442
$\beta^{\xi}_{FS_{survey\ period}}$	-1.139	0.442	-2.077	-1.111	-0.358
$\beta_{FH_{survey\ period}}$	-0.072	0.112	-0.293	-0.072	0.147
$\beta^{\xi}_{FH_{survey\ period}}$	-0.031	0.275	-0.579	-0.027	0.504
$\beta_{FS_{season}}$	-0.243	0.122	-0.483	-0.243	-0.003
$\beta^{\xi}_{FS_{season}}$	-0.443	0.372	-1.212	-0.432	0.260
$\beta_{FH_{season}}$	-0.064	0.110	-0.280	-0.064	0.150
$\beta^{\xi}_{FH_{season}}$	0.272	0.271	-0.260	0.271	0.809
$\beta_{PC_{season}}$	-0.544	0.103	-0.749	-0.545	-0.344
$\beta^{\xi}_{PC_{season}}$	-1.069	0.291	-1.678	-1.057	-0.529
$\beta_{PS_{season}}$	-0.584	0.161	-0.906	-0.583	-0.273
$\beta_{FH_{forb}}$	0.336	0.051	0.239	0.335	0.438
$\beta^{\xi}_{FH_{forb}}$	0.333	0.092	0.143	0.338	0.503
$\beta_{PS_{complexity}}$	-0.336	0.153	-0.637	-0.336	-0.032
$\beta_{PC_{complexity}}$	-0.273	0.103	-0.476	-0.273	-0.072
$\beta^{\xi}_{PC_{complexity}}$	-0.228	0.211	-0.640	-0.232	0.193
$\beta_{FH_{complexity}}$	-0.262	0.120	-0.494	-0.263	-0.025
$\beta^{\xi}_{FH_{complexity}}$	0.149	0.247	-0.341	0.151	0.632
$\beta_{FS_{complexity}}$	0.709	0.135	0.439	0.712	0.0.970
$\beta^{\xi}_{FS_{complexity}}$	0.960	0.291	0.404	0.954	1.556

Table 8: Correlation across years between park extinction and colonization rates: full model

	CRLA	CRMO	LABE	LAVO	NEWB
		extinction rate			
CRLA	1.00	0.22	0.35	0.25	-0.23
CRMO	0.22	1.00	0.61	-0.43	0.38
LABE	0.35	0.61	1.00	0.07	0.45
LAVO	0.25	-0.43	0.07	1.00	-0.15
NEWB	-0.23	0.38	0.45	-0.15	1.00
		colonization rate			
CRLA	1.00	0.56	0.31	0.56	0.57
CRMO	0.56	1.00	0.63	0.09	0.10
LABE	0.31	0.63	1.00	0.13	-0.24
LAVO	0.56	0.09	0.13	1.00	-0.03
NEWB	0.57	0.10	-0.24	-0.03	1.00

Table 9: 95% Credible intervals, standard deviation, mean of posterior estimates of region hyperparameter (η_r) influence on initial occupancy, colonization and extinction: full model

Park	mean	sd	2.5%	50%	97.5%
initial occupancy					
CRLA	1.757	0.854	0.010	1.788	3.367
CRMO	-1.213	0.878	-3.071	-1.156	0.364
LABE	-1.156	0.909	-3.069	-1.091	0.452
LAVO	-1.140	0.927	-3.116	-1.078	0.517
NEWB	0.513	1.726	-2.906	0.488	4.267
colonization rate					
CRLA	-0.640	0.867	-2.756	-0.521	0.754
CRMO	-1.472	0.933	-3.709	-1.362	-0.000
LABE	0.605	0.752	-1.015	0.621	2.003
LAVO	0.285	0.749	-1.448	0.315	1.654
NEWB	-0.155	0.834	-2.090	-0.075	1.311
extinction rate					
CRLA	-1.397	1.045	-3.649	-1.315	0.401
CRMO	-0.806	0.923	-2.931	-0.688	0.701
LABE	-0.982	0.925	-3.150	-0.871	0.532
LAVO	1.496	0.873	-0.188	1.484	3.242
NEWB	-0.223	0.879	-2.252	-0.146	1.339

Table 10: 95% Credible intervals, standard deviation and mean of posterior estimates of year within region hyperparameter (Y_{rt}) on occupancy processes: full model

Park	Year	mean	sd	2.5%	50%	97.5%
Colonization rates						
CRMO	2011	-2.343	1.305	-5.297	-2.213	-0.164
LABE	2012	1.747	0.946	-0.421	1.822	3.398
LAVO	2013	1.990	0.814	0.139	2.051	3.385
LABE	2014	2.351	0.870	0.440	2.393	3.920
CRMO	2017	-2.498	1.315	-5.461	-2.378	-0.288
CRMO	2018	-2.326	1.343	-5.333	-2.199	-0.022
CRMO	2021	-0.494	1.053	-2.812	-0.407	1.337
Extinction rates						
LAVO	2012	2.057	1.013	0.033	2.057	4.063
LAVO	2014	2.047	0.980	0.099	2.034	3.998

Table 11: 95% Credible intervals, standard deviation and mean of posterior estimates: non-false positive model

Parameter	mean	sd	2.5%	50%	97.5%
**overall occupancy dynamics/detection rates**					
Ω_{Ξ}	0.370	0.156	0.117	0.349	0.730
γ_{Ξ}	0.116	0.062	0.039	0.102	0.289
ε_{Ξ}	0.111	0.075	0.028	0.091	0.326
p_{fs}	0.699	0.018	0.664	0.699	0.733
p_{fh}	0.251	0.017	0.218	0.250	0.286
p_{pc}	0.385	0.016	0.354	0.385	0.417
p_{ps}	0.101	0.010	0.083	0.101	0.120
**occupancy process parameter estimates**					
$\alpha_{\Lambda_{complexity}}$	0.821	0.262	0.329	0.814	1.361
$\alpha_{\varepsilon_{forb}}$	-1.948	0.524	-3.098	-1.900	-1.065
$\alpha_{\varepsilon_{temp}}$	0.250	0.494	-0.709	0.254	1.219
$\alpha_{\varepsilon_{ppt}}$	-0.480	0.359	-1.274	-0.444	0.130
$\alpha_{\gamma_{forb}}$	0.191	0.103	-0.012	0.192	0.3904
$\alpha_{\gamma_{shrub}}$	0.243	0.101	0.048	0.242	0.447
$\alpha_{\gamma_{rock}}$	0.312	0.133	0.059	0.309	0.579
$\alpha_{\gamma_{temp}}$	-0.369	0.239	-0.852	-0.365	0.093
**observation process parameter estimates**					
$\beta_{FS_{survey\ period}}$	-0.629	0.095	-0.815	-0.629	-0.442
$\beta_{FH_{season}}$	-0.063	0.101	-0.261	-0.063	0.135
$\beta_{FS_{season}}$	-0.158	0.094	-0.340	-0.158	0.029
$\beta_{FH_{season}}$	0.031	0.099	-0.162	0.031	0.225
$\beta_{PC_{season}}$	-0.597	0.094	-0.779	-0.597	-0.414
$\beta_{PS_{season}}$	-0.542	0.157	-0.853	-0.540	-0.239
$\beta_{FH_{forb}}$	0.305	0.041	0.226	0.304	0.386
$\beta_{PS_{complexity}}$	-0.109	0.146	-0.394	-0.110	0.177
$\beta_{PC_{complexity}}$	-0.200	0.089	-0.375	-0.200	-0.024
$\beta_{FH_{complexity}}$	-0.110	0.106	-0.314	-0.111	0.101
$\beta_{FS_{complexity}}$	0.934	0.093	0.753	0.933	0.116

Table 12: 95% Credible intervals, standard deviation, mean of posterior estimates of region hyperparameter (η_r) influence on initial occupancy, colonization and extinction: non-false positive model

Park	mean	sd	2.5%	50%	97.5%
initial occupancy					
CRLA	1.834	0.769	0.246	1.846	3.373
CRMO	-1.183	0.782	-2.855	-1.141	0.299
LABE	-0.651	0.774	-2.309	-0.623	0.832
LAVO	-0.915	0.791	-2.591	-0.877	0.569
NEWB	0.106	1.955	-4.092	0.109	4.032
colonization rates					
CRLA	-0.726	0.724	-2.342	-0.659	0.526
CRMO	-1.334	0.719	-2.898	-1.283	-0.056
LABE	0.386	0.625	-0.917	0.391	1.616
LAVO	0.491	0.631	-0.807	0.494	1.741
NEWB	0.205	0.660	-1.169	0.210	1.517
extinction rates					
CRLA	-0.967	0.814	-2.783	-0.904	0.500
CRMO	-0.398	0.722	-2.034	-0.332	0.863
LABE	-1.055	0.747	-2.774	-0.975	0.204
LAVO	1.274	0.707	-0.173	1.276	2.669
NEWB	-0.160	0.726	-1.804	-0.104	1.159

Table 13: 95% Credible intervals, standard deviation and mean of posterior estimates: standard detection model

	mean	sd	2.5%	50%	97.5%
**overall occupancy dynamics/detection rates**					
Ω_{Ξ}	0.395	0.159	0.118	0.377	0.760
γ_{Ξ}	0.106	0.074	0.029	0.088	0.299
ε_{Ξ}	0.068	0.059	0.012	0.051	0.245
p	0.787	0.021	0.746	0.787	0.827
**occupancy process parameter estimates**					
$\alpha_{\Lambda_{complexity}}$	0.654	0.250	0.177	0.646	1.162
$\alpha_{\varepsilon_{forb}}$	-1.901	0.584	-3.144	-1.863	-0.869
$\alpha_{\varepsilon_{temp}}$	0.352	0.733	-1.017	0.351	1.795
$\alpha_{\varepsilon_{ppt}}$	-0.581	0.576	-1.840	-0.511	0.356
$\alpha_{\gamma_{forb}}$	0.146	0.123	-0.102	0.148	0.383
$\alpha_{\gamma_{shrub}}$	0.343	0.136	0.088	0.339	0.617
$\alpha_{\gamma_{rock}}$	0.359	0.172	0.035	0.352	0.710
$\alpha_{\gamma_{temp}}$	-0.433	0.290	-1.029	-0.426	0.126
**observation process parameter estimates**					
$\beta_{p_{complexity}}$	0.777	0.116	0.548	0.778	1.004
$\beta_{p_{season}}$	-0.561	0.113	-0.784	-0.560	-0.341
$\beta_{p_{survey\ period}}$	-0.586	0.119	-0.821	-0.586	-0.352
$\beta_{p_{forb}}$	0.374	0.071	0.239	0.371	0.519

Table 14: 95% Credible intervals, standard deviation and mean of posterior estimates: perfect detection model

	mean	sd	2.5%	50%	97.5%
**overall occupancy dynamics rates**					
Ω_{Ξ}	0.332	0.154	0.084	0.309	0.692
γ_{Ξ}	0.159	0.066	0.064	0.147	0.325
ε_{Ξ}	0.264	0.068	0.148	0.260	0.425
**occupancy process parameter estimates**					
$\alpha_{\Lambda_{complexity}}$	0.707	0.240	0.240	0.704	1.189
$\alpha_{\varepsilon_{forb}}$	-0.420	0.126	-0.679	-0.417	-0.185
$\alpha_{\varepsilon_{temp}}$	0.271	0.316	-0.309	0.276	0.869
$\alpha_{\varepsilon_{ppt}}$	-0.200	0.206	-0.616	-0.193	0.184
$\alpha_{\gamma_{forb}}$	0.276	0.078	0.125	0.276	0.432
$\alpha_{\gamma_{shrub}}$	0.198	0.073	0.055	0.199	0.339
$\alpha_{\gamma_{rock}}$	0.275	0.096	0.089	0.275	0.464
$\alpha_{\gamma_{temp}}$	-0.407	0.193	-0.793	-0.403	-0.034

Table 15: Posterior estimates for region random effect (η_r) influence on initial occupancy, colonization and extinction: perfect detection model

	mean	sd	2.5%	50%	97.5%
initial occupancy					
CRLA	1.791	0.802	0.190	1.790	3.464
CRMO	-1.057	0.817	-2.743	-1.025	0.568
LABE	-0.656	0.805	-2.302	-0.637	0.955
LAVO	-0.852	0.826	-2.559	-0.824	0.790
NEWB	0.071	1.885	-3.865	0.053	4.181
colonization rates					
CRLA	-0.433	0.595	-1.715	-0.403	0.656
CRMO	-1.296	0.599	-2.602	-1.263	-0.203
LABE	0.578	0.540	-0.506	0.572	1.666
LAVO	0.316	0.545	-0.802	0.323	1.396
NEWB	0.213	0.571	-0.940	0.209	1.381
extinction rates					
CRLA	-0.673	0.493	-1.665	-0.653	0.209
CRMO	-0.134	0.404	-1.009	-0.083	0.647
LABE	-0.207	0.406	-1.066	-0.168	0.562
LAVO	0.825	0.438	-0.000	0.818	1.769
NEWB	-0.057	0.415	-0.926	-0.019	0.767

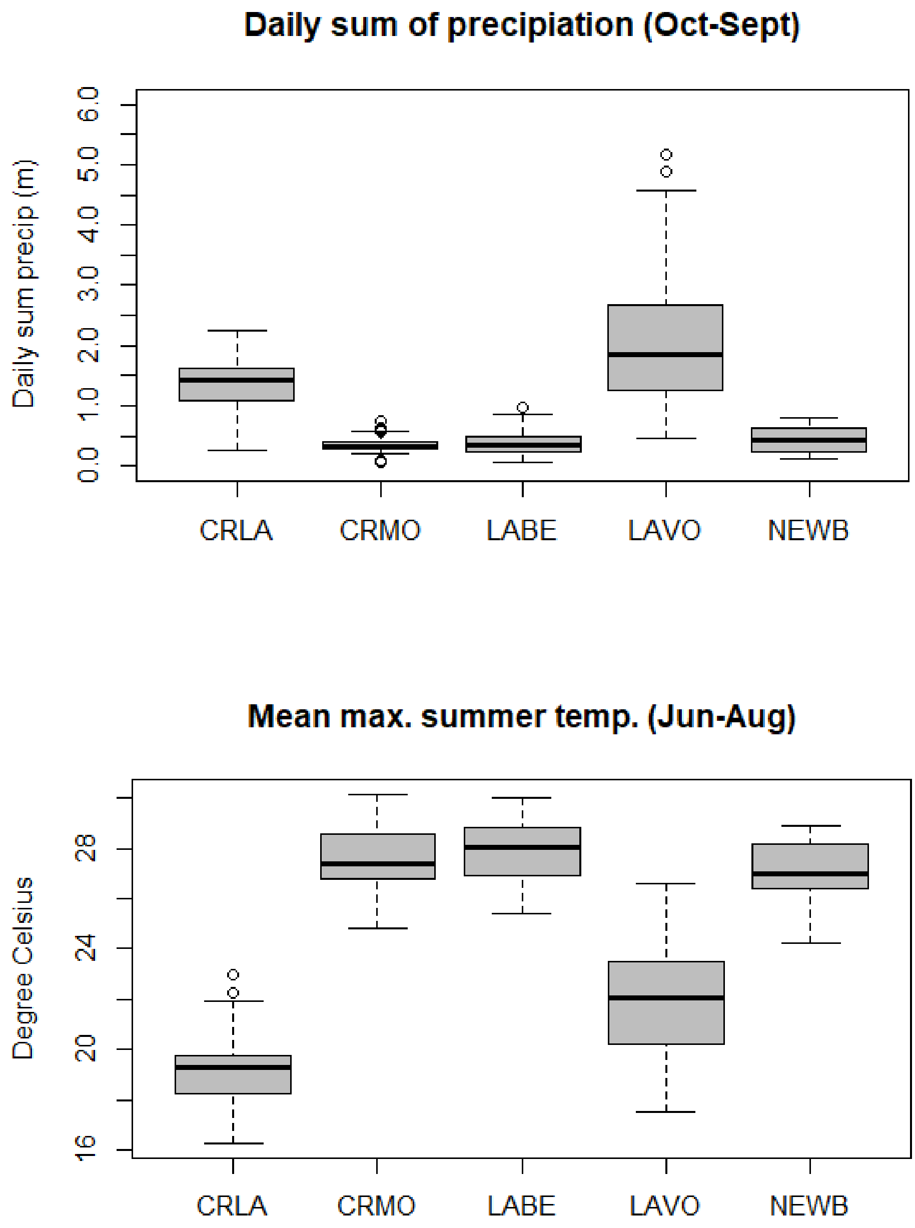

Figure 5: Top: Annual daily sum of precipitation (Oct-Sept, 2010–2021) across regions. LAVO has the greatest variation in precip. and highest annual precip (2.0m). CRMO and LABE indicate the lowest annual precip (0.4m and 0.5m). Bottom: Mean maximum summer temperature (Jun–Aug) across parks 2010–2021 where CRMO and LABE have the highest summer temp (27 °C and 28 °C) and CRLA has the lowest mean max. summer temps (18 °C).

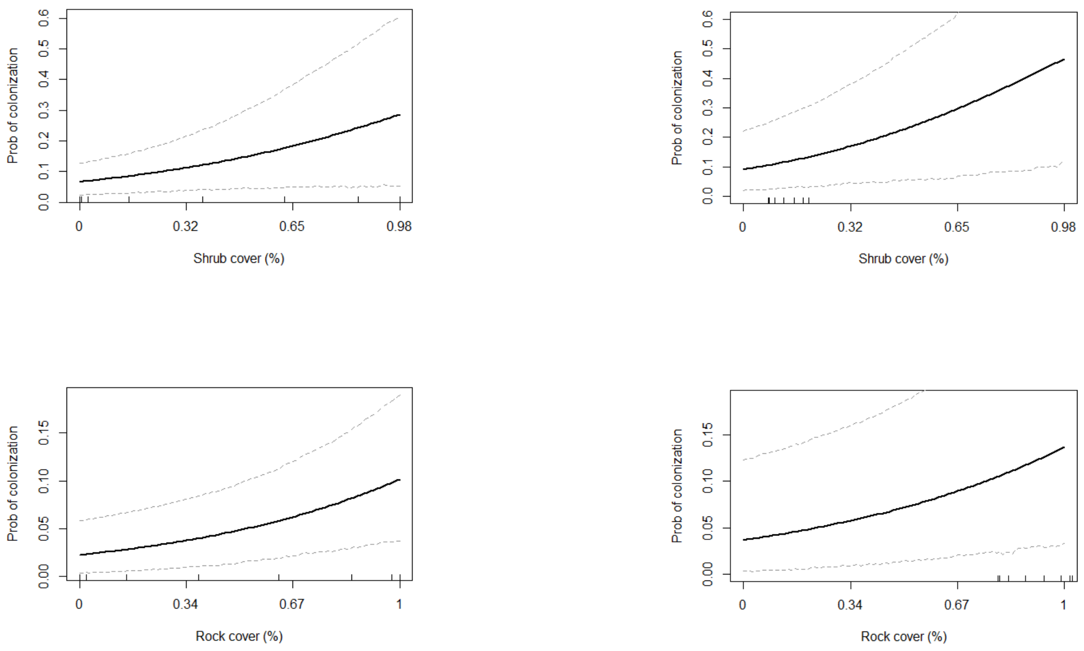

(a) full model

(b) non-false positive model

Figure 6: Linear regressions comparing the full and non-false positive model posterior estimate effects of shrub and rock cover on colonization rates across all parks. Dotted lines indicate 95% credible intervals.

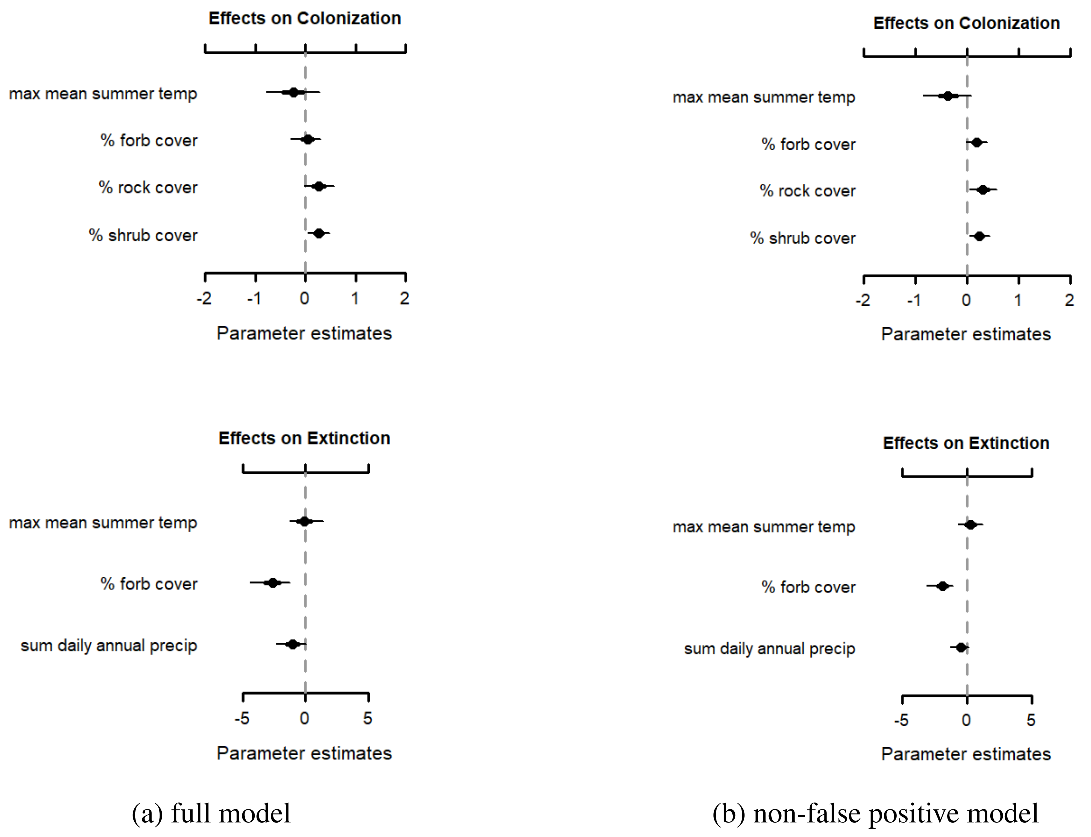

Figure 7: Comparison of full model and non-false positive model posterior estimate coefficient plots of environmental and climatic influences on extinction and colonization processes including mean maximum summer temperature, sum of daily annual precipitation and Daubenmire cover estimates across forb, rock and shrub cover.

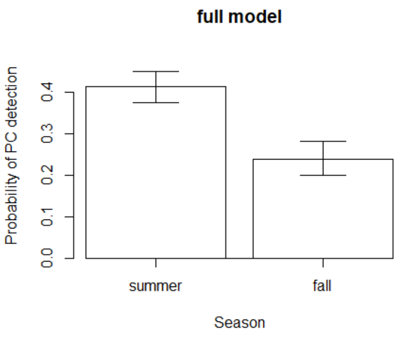

(a) Pika call

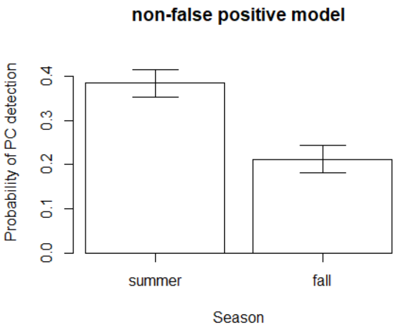

(b) Pika call

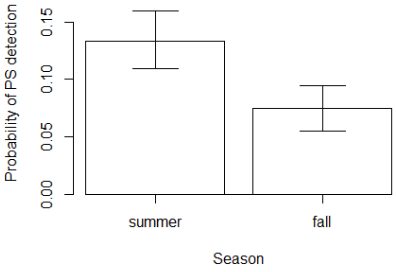

(c) Pika sighting

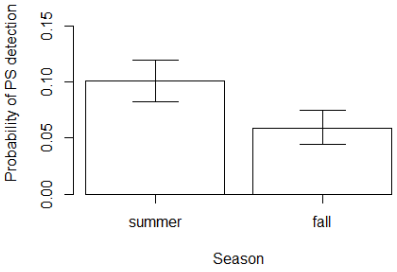

(d) Pika sighting

Figure 8: Bar plot comparison of full and non-false positive model parameter estimates of effect of season (summer or fall) on the probability of pika call and pika sighting detections across all parks.

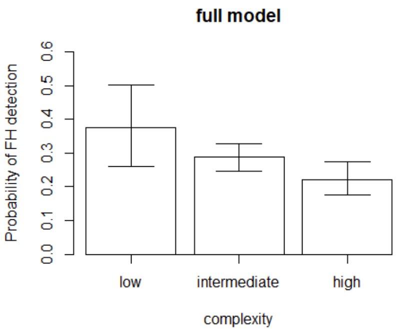

(a) haypile

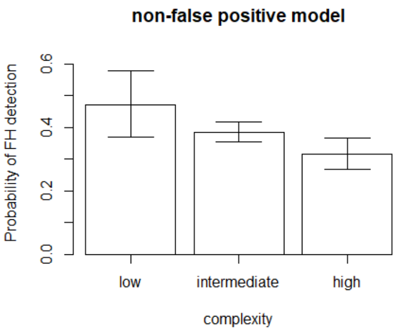

(b) haypile

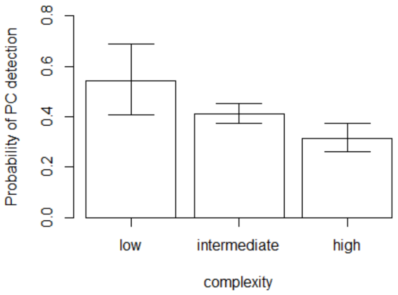

(c) pika call

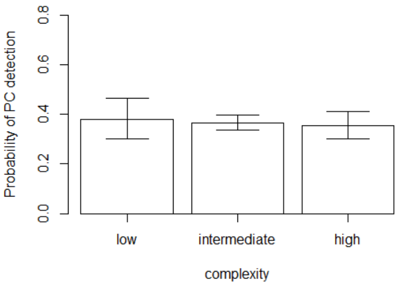

(d) pika call

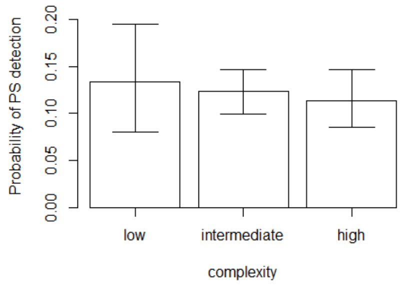

(a) pika sighting

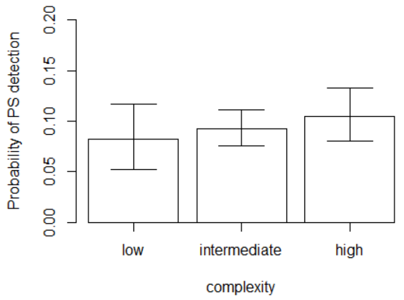

(b) pika sighting

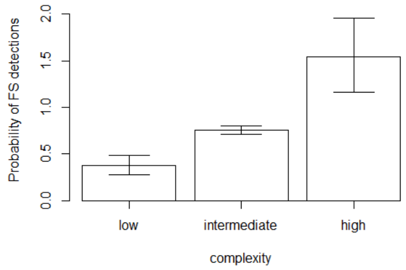

(c) fresh scat

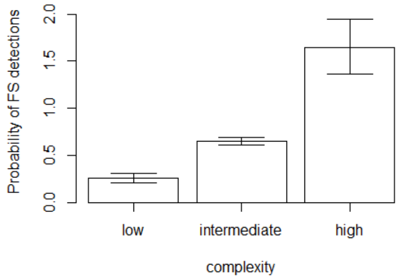

(d) fresh scat

Figure 10: Bar plot comparison of full and non-false positive model parameter estimate effect of rock complexity on the probability of all sign types across all parks.

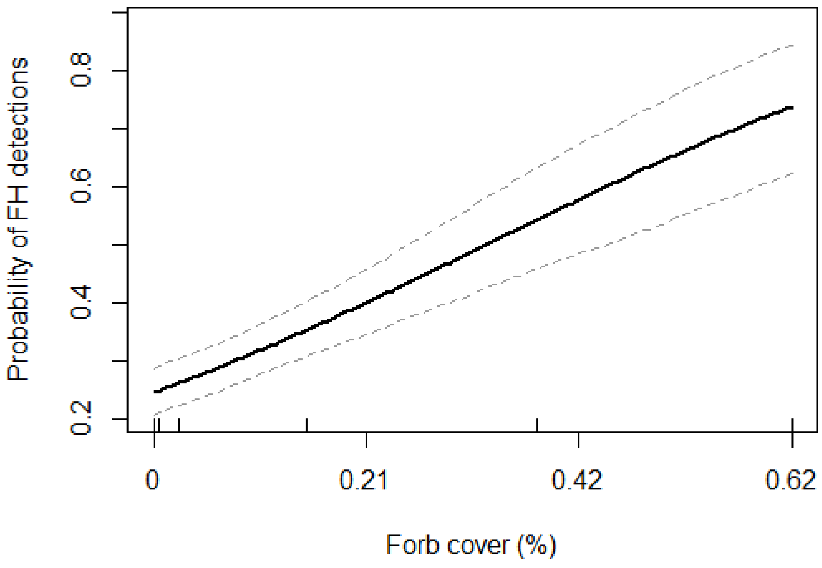

(a) full model: fresh haypile

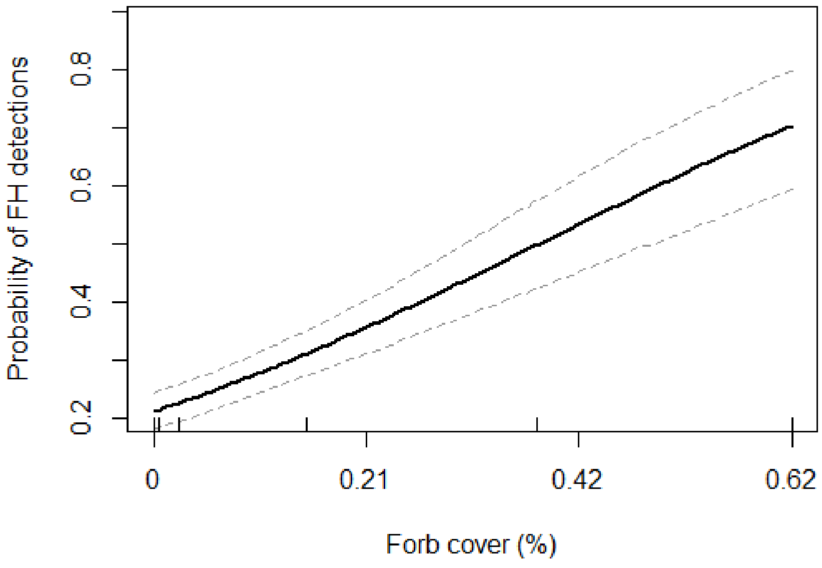

(b) non-false positive model: fresh haypile

Figure 11: Linear regressions comparing full and non-false positive model parameter estimate effect of percent forb cover on the probability of detection of fresh haypiles. Dotted lines indicated 95% credible intervals.

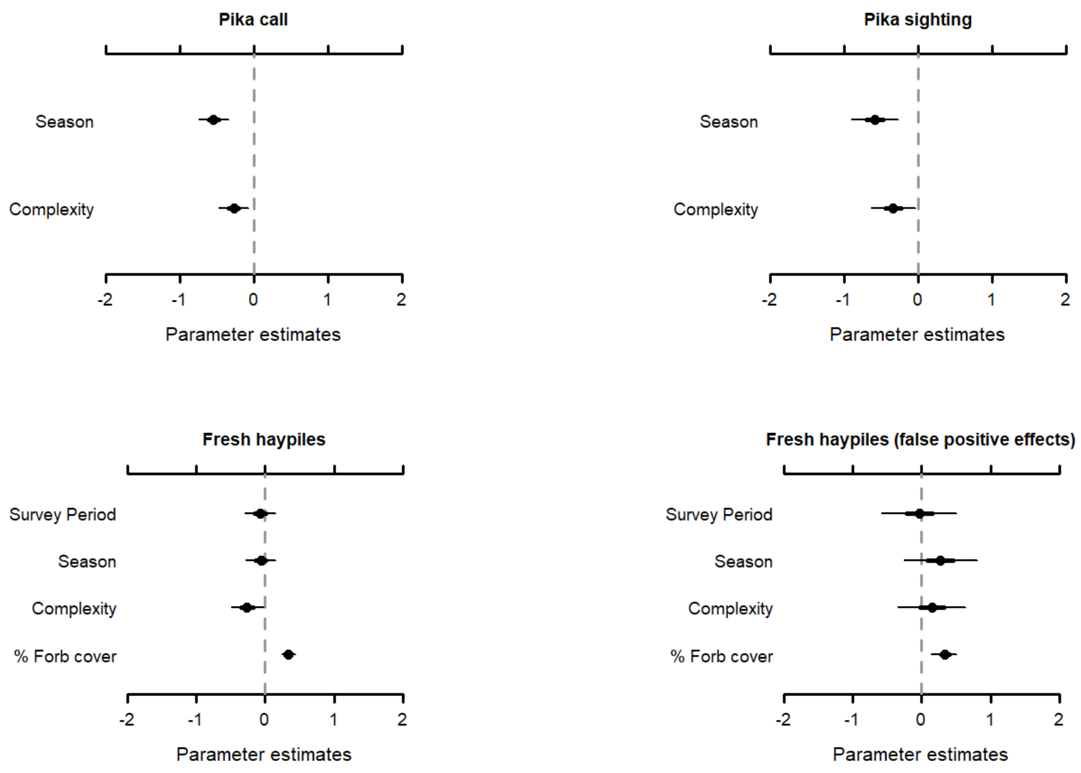

Figure 12: Full model coefficient plots of parameter estimates of the effects of season, complexity, survey period, and percent forb cover on pika call, sighting and fresh haypiles. Estimates indicated within 95% credible intervals.
